# Germinoma in the Internal Auditory Canal Mimicking a Vestibular Schwannoma

**DOI:** 10.1155/2014/794158

**Published:** 2014-01-06

**Authors:** Rubén Martín-Hernández, Diego Hernando Macías-Rodríguez, Ángel Muñoz-Herrera, Juan Carlos Del Pozo-de Dios, Santiago Santa Cruz-Ruiz, Ángel Batuecas-Caletrío

**Affiliations:** Skull Base Unit, ENT and Neurosurgery Department, University Hospital of Salamanca, Paseo San Vicente 58-182, 37007 Salamanca, Spain

## Abstract

The appearance of a primary germinoma in the central nervous system but not on or near the midline or within the brain is exceptional. It may occur at any age; however, it is rare in patients over 50 years old. Only a handful of cases of germinomas located in the cerebellopontine angle were presented, but to our knowledge, there has been no description of an isolated germinoma in the internal auditory canal. We report a case of germinoma in the internal auditory canal in a 51-year-old man simulating the clinical and radiological characteristics of a vestibular schwannoma.

## 1. Introduction

Extragonadal germ cell tumors located in the brain are rare and are most commonly found in the pineal and suprasellar region. Germinoma is the most frequent intracranial one. It has a good prognosis and response to treatment and a high incidence among the Chinese and Japanese population [1]. The combined incidence of central nervous system germ cell tumors in males of all ages was 3.7 times higher than in females [2]. The peak incidence of intracranial germ cell primary tumors is at puberty. Clinical signs and symptoms of intracranial germinomas include visual disturbances, delayed sexual maturity, diabetes insipidus, and growth retardation [3]. The diagnostic confirmation requires histopathological studies that usually show epithelial tumor cells with pale eosinophilic cytoplasm and large round nuclei with evident nucleoli, expressing variable and organized mitotic activity sheets, lobes, nodules, and cords separated by thin fibrous and vascular septa, with small lymphoid cells with a perivascular organization [1].

We describe a case of a germinoma in a male without any risk factor, located in a place where it has never been previously reported: the internal auditory canal (IAC). The patient had an unusual age for the appearance of a primary germinoma (51 years old). We observed a tumor that both clinically and radiologically mimicked a vestibular schwannoma.

## 2. Case Report

The patient is a 51-year-old man with right facial weakness of three weeks of evolution, including the inability to close the right eye (House-Brackmann grade 5/6 right facial weakness), bad response to oral corticoids, and deviation of the mouth on the same side. The patient did not present hearing loss, tinnitus, or ear fullness.

The pure tone audiometry demonstrated a mild bilateral sensorineural hearing loss, predominantly in high frequency tones. The tympanogram showed type A curves without ipsi- and contralateral blink reflex in the right ear. Otoacoustic emissions were absent in both ears. Brainstem auditory-evoked potential showed morphologically normal curves in both ears. The videonystagmography (VNG) did not detect spontaneous nystagmus and caloric tests were normal.

**Figure 1:**
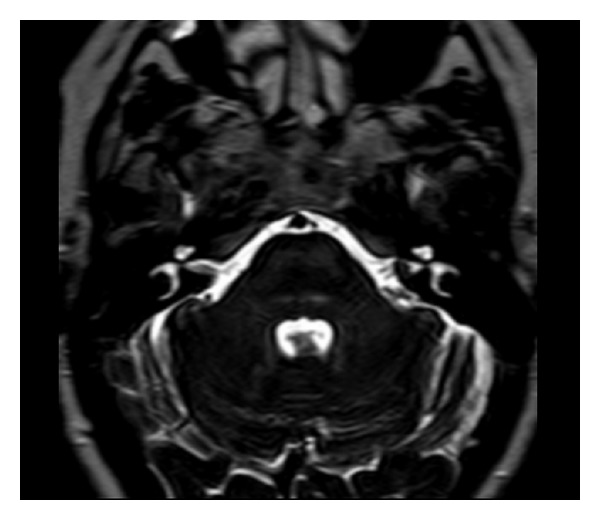
Mass of 0.8 × 0.4 mm into the right IAC.

The nuclear magnetic resonance (NMR) demonstrated a mass of 0.8 × 0.4 mm into the right IAC (Figure 1).

The patient underwent a total resection with a right retrosigmoidal approach to the cerebellopontine angle (CPA). Intraoperatively, we found that the tumor involved cranial nerves VII and VIII.

**Figure 2:**
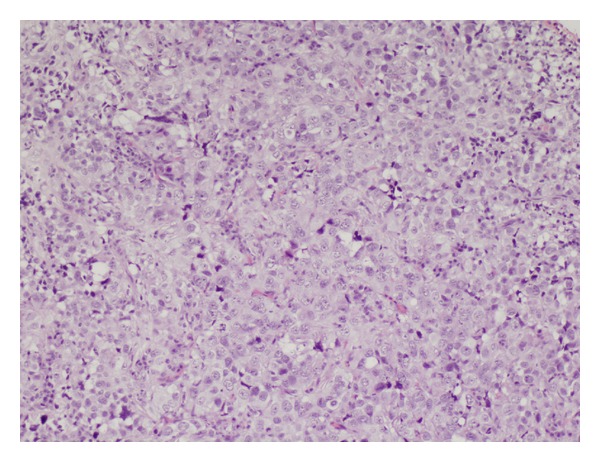
The microscopic examination shows irregular structure comprising cords of epithelial-like cells with a large nucleus, vascular connective stroma, and leukocyte infiltrates of variable density.

Microscopic examination revealed histopathologic findings of germinoma (Figure 2).

It shows an irregular structure comprising cords of epithelial-like cells with a large nucleus. Nucleoli are evident and there are a clumped chromatin with occasional mitotic figures, between which there is a scarce vascular connective stroma and leukocyte infiltrates of variable density.

In the immunohistochemical study, the cellularity was positive for cytokeratins of low, medium, and high molecular weight and for cytokeratins AE1, AE3, and EMA. Vimentin was also positive. The PLAP provides small foci discontinuous positivity and lymphocytic cellularity was predominantly positive for CD43 and to a lesser extent for CD20. B-HCG was negative. Ki67 was positive in 15–20% of the cells. GFAP, neurofilament, and ENE were negative.

The described data, both morphologically and immunohistochemically, are compatible with germinoma.

Concerning audiovestibular symptoms, our patient showed dizziness for three months after surgery because the auditory nerve was not preserved.

Further research for the primary tumor site (including positron emission tomography) was negative in all cases.

## 3. Discussion

Medical Subject Headings (MeSH) has defined germinoma as “a malignant neoplasm of the germinal tissue of the gonads, mediastinum or pineal region” [4, 5]. Germ cell tumors in the central nervous system (CNS) affect children and adults, and they appear predominantly in the first and second decade of life; the peak incidence is reached at 10–19 years of age. Age distribution of CNS germinomas is as follows [2]:

- 0–14 years: 34% of cases,
- 15–29 years: 57% of cases,
- 30–44 years: 9% of cases.

Otherwise, vestibular schwannomas and meningiomas represent more than 95% of the CPA neoplasms [6, 7]. There are other less common lesions such as lipomas, arachnoid cysts, hemangiomas, choroid plexus papillomas, metastatic disease, and collision tumors [7].

Anyway, germinomas in people over 50 years old are rare, germinomas affecting the CPA are extremely rare, but and germinomas in the IAC have never been reported.

Metastases and extra-axial primary malignancies are often initially misdiagnosed as benign disease [6] due to the fact that physical examination, clinical history, and audiovestibular testing are often unhelpful in discriminating the type of lesion because the involvement of CPA tumor is similar to a vestibular schwannoma with hearing loss, tinnitus, facial weakness, and disequilibrium [8].

Adhesion of the tumor to cranial nerves and surrounding tissues at surgery suggests uncommon tumor entities [7].

## 4. Conclusions

Clinical and radiological signs for CPA and IAC lesions are nonspecific. A final diagnosis can only be established based on histopathological analysis [7].

Although malignant tumors rarely appear in the CPA and IAC, they need to be included in the differential diagnosis. In patients without known malignancies, short duration of symptoms and rapid progression suggest malignant tumor entities [7]. In our opinion, patients with tumors affecting IAC should be warned about the possibility that they could not be benign tumors, especially when facial weakness is the main symptom [9].
